# Psychotherapy as making

**DOI:** 10.3389/fpsyg.2022.1048665

**Published:** 2022-11-17

**Authors:** John McLeod, Rolf Sundet

**Affiliations:** ^1^Institute for Integrative Counselling and Psychotherapy, Dublin, Ireland; ^2^School of Applied Science, Abertay University, Dundee, United Kingdom; ^3^Department of Health, Social and Welfare Studies, Faculty of Health and Social Science, Centre for Mental Health and Substance Abuse, University of South-Eastern Norway, Kongsberg, Norway

**Keywords:** action language, co-production, hylomorphism, improvisation, interdisciplinary perspectives, meta-theory, psychotherapy, training

## Abstract

Historically, research and practice of psychotherapy have been conducted within conceptual frameworks defined in terms of theoretical models. These models are in turn guided by meta-theories about the purpose of psychotherapy and its place in society. An image of psychotherapy that underpins much contemporary practice is the idea that therapy operates as an intervention that involves the implementation and application of a pre-existing theoretical model or set of empirically validated procedures. The present paper introduces the idea that it may be valuable to regard psychotherapy not as an intervention but instead as a process of making, in the sense of offering a cultural space for the co-construction of meaningful and satisfying ways of living that draw on shared cultural resources. We offer an overview of what a therapy of making might look like, followed by an account of theoretical perspectives, both within the psychotherapy literature and derived from wider philosophical and social science sources, that we have found valuable in terms of making sense of this way of thinking about practice. Our conclusion is that we need something in addition to theory-specific and protocol-driven therapies, in order to be able to incorporate the unexpected, the not-before-met perspective, event or practice of living, and to be open towards the radically new, the given, and the unknown.

## Introduction

Historically, research and practice of psychotherapy have been mainly conducted within conceptual frameworks defined in terms of theoretical models such as those provided by psychoanalysis, CBT, narrative therapy, person-centered therapy, and other approaches (McLeod, 2019). Such theoretical models are powerful. In assembling concepts, research evidence and practical skills into coherent packages, each therapy approach provides a coherent standpoint that enables the practitioner to facilitate change in the behavior or psychological functioning of the client or patient.

Approaches to therapy are guided by meta-theories - implicit images, metaphors, discourses and perspectives that reflect assumptions about the purpose of psychotherapy and its place in society, and operate as points of meeting and connection across theoretical approaches and areas of specialism (Najavits, 1993; Najavits, 1997). The image of psychotherapy that underpins much contemporary practice is an idea of therapy as an *intervention* that involves the application of a pre-existing theoretical model or set of empirically validated procedures.

Describing psychotherapy as an intervention is commonplace across the contemporary psychotherapy research and practice literature. For example, the term is widely used in psychotherapy studies and reviews in relation to such approaches as cognitive-behavioral therapy (Keles and Idsoe, 2018; Miller et al., 2021), psychoanalytic psychotherapy (Jones et al., 2020; Pitillas, 2020) and family therapy (Tsvieli and Diamond, 2018; Carr, 2019), as well as in cross-theoretical articles (Lamb et al., 2019). At the present time, one of leading journals in the field of psychotherapy, the *Journal of Clinical Psychology,* routinely includes a sub-section titled “Intervention Research.” Although the concept of intervention is often associated with a medicalized form of practice in which diagnosis leads to the implementation of an evidence-based intervention, such an understanding does not, in our view, do justice to the multi-faceted nature of medical practice. For example, there are many situations in medical practice where standardized interventions are not available (Eriksen et al., 2013; Husain and Chalder, 2021). In addition, studies that have analyzed the real-world decision-making processes exhibited by physicians take account of many factors beyond the selection and application of a surgical or pharmacological intervention (Gabbay and le May, 2010). Our account of the limitations and implications of viewing psychotherapy as an intervention is not intended to represent a challenge to the potential relevance of medical and biological perspectives to psychotherapy practice. Rather, drawing attention to the taken-for-granted use of the concept of intervention invites consideration of important aspects of this meta-theoretical construct that are rarely considered, such as an assuming that the capacity to bring about change ultimately resides in professional knowledge and expertise, rather than being grounded in an emergent collaborative process that draws on the knowledge and experience of the client (Dreier, 2011).

The present paper uses critical conceptual analysis, and examples from personal experience and practice, to explore the possibility that it may be valuable to regard psychotherapy not as an intervention, but instead as a process of *making,* in the sense of offering a cultural space for co-producing satisfying ways of living that draws on shared cultural resources. We offer an overview of what a therapy of making might look like, followed by an account of theoretical perspectives, both within the psychotherapy literature and derived from wider philosophical and social science sources, that we have found valuable in terms of making sense of this way of thinking about practice.

## Psychotherapy as a process of making

The idea that psychotherapy can be regarded as a form of making refers to the general human understanding of making as involving purposeful activity to create some kind of object or event that can then be referred to as a discrete entity. An essential aspect of any type of making is that it requires a person or persons with a sense of wanting to achieve something, who then engage in a process that involves many steps. Making can be contrasted with a process of joining together pieces of an object in accordance with a pre-determined set of instructions: a genuine experience of making always has an emergent quality in which each step creates a context that leads to further choices. Everyday making may involve a lengthy and multi-faceted sequence of activity such as making a home for oneself or may refer to a more discrete activity such as making a meal. The significance of making as an aspect of everyday life is reflected in an extensive lexicon of ‘making’ words, such as constructing, building, creating, producing, shaping, crafting and repairing, as well as phrases such as making it, making up, making out, and making amends.

Thinking about psychotherapy as making leads to the question of what are the basic ingredients or elements from which the eventual products of therapy are constructed, how this process unfolds, and what it is that is eventually made. The basic ingredients of therapy consist of everyday language, cultural practices, and a capacity for emotional presence and attunement. A key element is talk: everyday, non-technical language provides a rich resource for about relationships, emotions and life goals, along with ways of adopting alternative positions (I, we, you, they, and it). All cultures offer resources for expressing, collaborating on, and transforming problems in living: story structures, interaction rituals, music, art, drama. Being human means having access to an array of strategies for making, re-making and repairing lives. These tools, strategies and knowledges are available to the client and therapist, and represent the common ground on which they meet. In addition to these shared general cultural resources, therapist and client possess their own personal ideas, templates, theories, preferences or recipes around how personal learning, growth, change, recovery, or healing can be accomplished.

The existence of a cultural reservoir of common knowledge about how to handle problems in living is supported by multiple strands of evidence (McLeod and McLeod, 2022). One particularly important body of research is represented by the work of Tomasello (2015), who investigated human capacities for collaboration and sharing of emotional states and intentions, as evolutionary mechanisms that operated as drivers of human survival in pre-history and still exist within our bodies and nervous systems today. Other evidence comes from sociological and social anthropological research into the expression of caring and compassion within ordinary lives. An example of such research can be found in the work of Brownlie (2014) and Brownlie and Anderson (2017) who used interviews, diaries and analysis of social media to document the importance for individuals of shared activities, spontaneous acts of kindness and generosity, and a capacity to “be there” for each other. A final domain of inquiry around common knowledge of how to respond to problems in living, comprises a body of research into the wide range of non-medicalized, culturally embedded activities (e.g., adjusting diet, spending time with pets, making to-do lists, engaging in pleasurable distractions, etc.) that individuals are aware of, and implement, when confronted by problem in living (Jorm et al., 2004; Villaggi et al., 2015; van Grieken et al., 2018).

At the present time, both theoretically-driven and empirically-validated interventionist models of psychotherapy operate from a position of superimposing a pre-determined set of ideas and procedures on the messy and troubled life situation presented by the client, and use standardized interventions, consistent with the theory being implemented, to bring about improvement or cure of the patient’s condition. By contrast, a therapy as making perspective takes as its starting point the idea that *both* client and therapist are in possession of knowledge and experience that is potentially relevant and helpful to the messy and troubled life of the patient, and that the best way forward is to work together to make (in the sense of co-producing) some kind of new way or arranging the parts of that life into a form that is more satisfying for the person and their family.

It is important to acknowledge that all psychotherapy incorporates elements of making and intervening (as well as drawing on other guiding metaphors such as learning and growing). However, at the present time the dominant tradition is interventionist: the most influential psychotherapy theories, research programs and training models are grounded in an underlying position of intervening, situated in institutional contexts (e.g., medical and State-funded) that are permeated by an interventionist ethos. It is therefore hard for any therapist (including ourselves) to avoid drawing on intervention metaphors when working with clients. However, we believe that, even in the most manualized and empirically validated therapies, there are still many threads of the work that are improvised and co-produced, reflecting a process of weaving, making and co-creating something that is locally and contextually appropriate. In advocating greater attention to psychotherapy as making, we are drawing attention to a long established (but somewhat marginalized) counter-tradition of practice represented by such figures such as Anderson (2007), Bohart (2015), Haley (1973), Shotter (2010) and Smedslund (2012), Smedslund (2016), and the Open Dialogue approach by Seikkula and Arnkil (2017).

## The emergence in our own practice of an understanding of psychotherapy as making

To illustrate what a therapy of making might look like in practice, we offer a series of episodes from our own lives and professional careers that have represented key learning points in our development of this perspective.

### Finding a way of working with clients who do not conform to theoretical assumptions

In 1989 I (RS) started to work in an outpatient clinic within the mental health service for children and adolescents. Much of the therapy I provided was with young boys classified by the system as unmotivated and not fit for psychotherapy. These boys were all different. If there was a general characteristic it was one of reluctance, of not openly refusing to attend, but never taking a clear position on wanting to be in the session. They were sent by their parents, school and/or primary physician because they were a source of concern for the adults. They could be given different diagnoses, often some behavioral description of not attending to school work, being seen as unmotivated for anything but avoidance and mischief (Sundet, 2004).

On arriving they usually studied the floor, answered my questions with as few words as possible, showing no clear interest and belief in what a psychologist had to offer, at the same time not being offensive. They communicated through their non-verbal language that they were there in order to get out as quickly as possible, at the same time showing signs of resignation given that authorities had told them they needed to see a psychologist. One boy had been referred because he hit his mother. His smile was friendly, he was polite, and replied “I do not know” when asked about his problem and what he thought about having been referred to the outpatient clinic to see me. Another frequent answer was “It’s all the same with me.” This was not the starting point that I had been trained to have as a psychologist, but I had to take what I got, so what happened?

The key experience that changed my life as a therapist was discomfort. The discomfort became so great meeting these boys` pain in being together with me that I started to do things that had not been recommended in my training. I started to avoid theoretically-informed questions (“can you tell me about the incident at school?”), and themes (“could you tell me about your relationship with your parents/teachers?”). I became personal and started to tell clients about myself and my family (“I have a stepson at your age. He also does skateboarding”) and my interests (“I love western movies and movies about Dracula”). I started to invite them to tell and show me what they were doing in their spare time; how to do a “tricks” on their skateboard, how high they could do a karate kick, how to fake good results at school. Over time a way of being together developed where these boys came back for further sessions. The discomfort subsided, and I started to experience other feelings in the meetings: vitality, humor, interest and communality - all based on replacing traditional “therapist behaviors” with the intention of being personal and attentive to joint areas of focus and interest, learning about their ideas for making their lives different. I began to experience a lessening in the problems these boys were referred for in reports from their parents and teachers. It seemed to me that one of the main ways in which therapy had been helpful was to offer these boys an experience of making a relationship with an adult in which their own experience and capabilities were taken seriously, something that they might be able to create in relationships with other adults.

### Being disappointed by therapy that did not allow space to make what i wanted to make

I (JM) was seeing a therapist in the hope of learning how to cope with an increasing intensity of stress, anxiety and physical symptoms. My therapist had been trained in a humanistic, experiential approach that encouraged awareness of what was happening in the present moment. Attending therapy sessions had the effect of providing a regular weekly space in a crowded diary, where I could talk about whatever was concerning me. Initially, I found this helpful. In retrospect, I think that what made it useful was the routine of taking a few hours for myself (not just the therapy session but travelling time), and the consistent reminder that there was more to my life than professional roles and tasks. A few weeks into therapy, I had a sense that I could go further. I came up with an idea that it would be useful to close my diary to new commitments for a period of three months, complete the massive backlog of work that constantly burdened me, and see what would happen. The only way I could envisage myself following-through with this plan was to use the weekly therapy sessions as a source of support. I was excited but also apprehensive about this whole project, because it represented a major personal experiment and venture into unknown territory. It never happened. When I mentioned the idea to my therapist, she was not interested. I do not recall the details of the conversation we had. It was not a long conversation. I felt hurt and diminished. Somehow it was not the kind of thing that my therapist regarded as a relevant use of her time. I had a sense that she believed that I was avoiding something.

### Learning to help clients whose lives had not been improved by previous interventions

In 2001 RS started to work at the Family Unit, a combined day-treatment and outpatient family unit within the Department of Child and Adolescent Psychiatry in a Norwegian hospital. This unit received referrals from general practitioners, school and child protection services. It was serviced by five therapists with a residential apartment at its disposal. The unit offered outpatient treatment combined with a possibility of staying in the apartment for three weeks. Often the families had tried other treatment programs without success. They recounted experiences of not being listened to and heard by prior therapists, often connected to bureaucratic “rules” that specified how therapy should be done. These did not fit with the experiences and perspectives of the family. In response to these prior experiences, we developed a principle of always trying to “follow the family.”

An example of this can be found in our meetings with a family with a young girl in distress, where dealing with fatigue and other serious somatic symptoms had become the daily reality for her and the family. No physical cause was found for these states. The conclusion was a diagnosis that highlighted a psychological etiology. The therapeutic idea in prior treatments had been to not give in to these symptoms, but continually challenge them, keep the pressure up and at the same time attend family sessions where family relationships were investigated. The family experienced that everything continued to get worse. As the problems worsened, so did the therapeutic relationship. In the end everyone involved said “stop” and the family were referred to the Family Unit. The principle of following the family was attended to by literally doing what the family said needed to be done by applying continuous monitoring of process and outcome feedback. Following the feedback was the starting point for conversations around what might be helpful, and how we might work together.

What did this family tell us? To slow down the pace, ease up and allow their child to decide how much activity to engage in during the ordinary pursuits of the day, expressed through the statement “take away all pressure.” They asked the therapists to have faith in the process, their child and in the ability of everyone to find a way. Our job became to provide authority for, and acceptance of, such a strategy in relation to health authorities, the school and groups of professionals that was involved. The whole family expressed an expectation of the therapists to actively take part in fighting any perspective that turned her condition into a psychological and psychiatric concern. They asked us to support them in making a space where they could tackle the problem in their own way. Where did this end? Over time, the girl and her family reported that mood, vitality and participation in daily life increased for all of them. They found ways to live with the condition and its effects, by making adjustments to daily routines that allowed them to have a more satisfying life together, and a return to school.

### How Alec used therapy to make a new life

Alec was a man in his late twenties who came to see me (JM) in my private practice because his life had collapsed around him. He was employed in what he viewed as a dead-end job that did not match his interests and abilities. Panic attacks, depression, and crippling self-doubt were threaded through his day-to-day experience of life. He described himself as having reached rock bottom, and felt as though he needed to re-make his life. Alec responded positively to my suggestion that we might work together to find specific tasks that would begin make a difference. We created a visual map that provided a basis for understanding of how his problems had developed, how they were triggered, and means through which his life goals might be achieved. In the following weeks Alec built up and tried out a set of strategies for handling or avoiding panic attacks, partly based on CBT principles and partly on his own appreciation of what he had found helpful in the past. He initiated activities that supported positive well-being through re-establishing time with friends through sport and walking in the country. He discovered that – to his surprise - these friends turned out to be supportive and understanding when he told them about his panic attacks and depression. They told him about problems in living that they themselves had encountered, and had ideas about how to manage. Alec decided that the time had come to repair his relationship with his father, and – with his father’s help – began to engage in a systematic search of career options and explore the viability of specific career choices. Over the course of four months of weekly individual therapy Alec made substantial progress in relation to making sense of traumatic events that had occurred in early childhood, and beginning a process of moving forward in his life and making up for lost time.

These anonymized fragments of therapy practice have been selected to convey some of what we regard as key aspects of a view of therapy as making: open-ness to the initiatives and knowledge of clients, willingness to make use of our own personal in-the-moment responsiveness as well as our therapy theories, a commitment to working together in the sense of taking turns to lead and follow, and regularly taking stock of progress to ensure what we were making together was aligned with what the client wanted from therapy.

## Building a conceptual framework to support a therapy of making

The notion that psychotherapy can be understood as a form of making represents a particular image, metaphor or meta-theory that can be contrasted with other metaphors for therapy (Najavits, 1993). As with any metaphor, the idea of making highlights certain aspects of a phenomenon or activity, while downplaying other aspects. It also operates discursively, as a way of talking about an area of experience that positions the speaker in relation to wider political and moral debates and traditions within a culture. The meaning of a word like ‘making’, that refers to a fundamental and universal aspect of functioning and surviving as a person and human being, is complex, and open to different interpretations that shift in response to change in society. At the present time, the concept of making operates at a taken-for-granted level within everyday life (“I made a meal,” “he made a mess”). However, it has also come to signify a distinctive philosophical, moral and political stance. To talk about psychotherapy as making is to invoke a particular way of seeing therapy, that invites connections to be made with important perspectives and lines of thinking within contemporary culture, that exist beyond the field of psychology and psychotherapy.

In the following sections, we offer a brief introduction to some of the perspectives that have contributed to our own understanding of psychotherapy as making. It is important to be clear about the function and status of these ideas. We are not suggesting that approaching therapy as a context or opportunity for making, is determined by, or an application of, an underlying philosophical position or theory. What we are seeking to establish, instead, is that if therapy is understood as a practical activity in which two (or more) people work together to make (repair, build) some aspect of social life, then there are certain ideas and concepts that are helpful in relation to the task of making sense of what they are doing. Another way of putting this is that ways of making sense of making that have been formulated by those in other disciplines have the potential to enable us to be better therapists and clients, and that dialogue across these intellectual and professional boundaries has the potential to be mutually beneficial. There are three perspectives on making that we have found particularly valuable: undoing hylomorphism, co-producing, and making as connecting.

### Undoing hylomophism

A considerable amount of research by archaeologists, social anthropologists and historians has focused on the origins and evolution of the human capacity to make things, such as tools and shelters, and how ways of understanding making have changed over time. Within human culture, early tool-use and capacity to make things were grounded in manual and embodied action: shaping and combining whatever materials were available in a manner that took account of the specific characteristics of the local situation. The earliest humans engaged in making tools out of bone and stone, making pots out of clay, and using fire to cook and to control aspects of their environment. The development of weaving introduced wider possibilities for making, through its requirement to develop an awareness of lines (roots, branches, grass fibers), and the development of advanced skills such braiding, knotting and repairing, necessary in order to create larger structures. Alongside a growing ability to make things, there emerged a capacity to communicate and share ideas and skills.

These modes of making represent fundamental aspects of human existence that continue to underpin all domains of social life, as traditional “knowing-how,” where the maker works with whatever is at hand, using practical skills that are passed on from one person to the next. Historically, this kind of improvised, practical making was something that people just did – it was not guided by any overarching theory of how it operated. Gradually, however, it began to be understood in terms of a philosophical perspective known as *hylomorphism* (Ingold, 2010). The concept of hylomorphism is concerned with the question of how to make sense of the consistency and change that we observe in the world around us. Aristotle argued that it was necessary to make a distinction between form and matter: while each plate or beaker produced by a mold consists of a different set of particles (matter), we recognize that they are all share the same form. This idea then inevitably leads to an assumption that knowledge and wisdom depend on the ability to identify underlying forms or structures that lie behind what is immediately observable.

Transferred to the realm of practical activity, hylomorphism and the distinction between form and matter leads to the idea that the making process is ultimately determined and guided by a pre-existing abstract idea that exists in the mind of maker. The concept of hylomorphism is deeply rooted in Western civilization. The historian, social anthropologist and political philosopher James C. Scott (2008), Scott (2017) argues that human society shifted, over the course of thousands of years, from a largely egalitarian mode, within small bands or family groups, through agrarianism and farming, to its present organization in terms of nation states. For Scott (2008), a key process within this historical transition concerned the way that the adoption of an agrarian way of life (farms, then villages, then cities) made it possible to support a class of people (rulers, priests, philosophers) who stood outside of the activities of food production and were able to generate abstract ideas, theories and ideologies about how life should be lived, that they then had the power to impose on the population in general.

In relation to making, this meant that the status of practical making, guided by embodied knowing and the use of the hand, was gradually diminished:

…in the history of the Western world (…) the tactile and sensuous knowledge of line and surface that had guided practitioners through their varied and heterogeneous materials, like wayfarers through the terrain, gave way to an eye for geometrical form, conceived in the abstract in advance of its realization in a now homogenized material medium. …the …physical and material basis of making has been progressively devalued, while the hylomorphic model has gained in strength. (Ingold, 2010, p. 92–93).

Ingold (2013) suggests that a significant example of a shift in the balance between hylomorphism and emergent making can be identified in 15th century historical sources that document the transition from cathedrals being built by master craftsmen who adapted their materials and techniques to the specific task in hand (most of the great cathedrals of Europe were constructed in this manner), to the emergence of “master-mind” architects who drew up detailed plans in advance (Ingold, 2013; Gürsoy, 2016).

Within Western thought, the domination of a hylomorphic stance was further reinforced by the dualist philosophy of Descartes that emphasized the primacy of mind and thought, and the emergence of a science that sought to establish abstract, mathematically-definable laws of nature as introduced by Isaac Newton (Toulmin, 1992). Graeber and Wengrow (2021) have argued that, while Eurocentric examples of hylomorphic thinking have received most attention, these tensions have existed across all of human history and pre-history, in the form of evidence of oscillation between local, collective decision-making, and hierarchical and centrally-controlled forms of life.

Hylomorphism reflects an approach to making that starts with an idea or mental image and from this implements a process that ends with the finalizing of an object, piece of art or product that is a realization of the original idea or image: “whenever we read that in the making of artefacts, practitioners impose forms internal to the mind upon a material world ´out there`, hylomorphism is at work” (Ingold, 2013, p. 211). In meeting the material, the maker shapes a product through actions that are in accordance with a pre-existing idea or theory. There is a distance between the maker and the material; the maker stands on the outside, imposing their ideas on the material. With the hylomorphic model:

“…an agent with a particular design in mind, answering to his or her purpose, while matter – thus rendered passive and inert – became that which was imposed upon” (Ingold, 2013, p. 27).

“…the maker begins with both a plan and a finite set of component operations required to implement it. As the task proceeds, these components are assembled, bit by bit, to constitute a totality that corresponds precisely to the original design. But only with the last operation … does the artefact come into its own as coherent work. … (There can) … be no final product without an initial design, no completion without an origin. For finality can only be judged in relation to a project of assembly that is already prefigured at the outset, albeit in virtual form, in the mind of the maker” (Ingold, 2013, p. 41)

Contemporary evidence-based psychotherapy can be viewed as primarily operating on the basis of a hylomorphic perspective: the characteristics of hylomomorphism outlined above can be applied to any therapy approach that is guided by a specific theory or treatment manual.

Our account does not do justice to the extensive philosophical literature around the concept of hylomorphism (Barnes, 2003; Manning, 2013). A key thread in this debate has been the argument that some version of hylomorphism seems to comprise a necessary element of modern science. Nevertheless, despite the centrality of hylomorphism within the ontology of contemporary society, there is a growing critical literature that views it as an unsatisfactory way of explaining human action and making (Ingold, 2010; Ingold, 2013; Malafouris, 2013; Malafouris, 2019; Malafouris, 2020; Griffiths, 2021). An alternative position is a return to a notion of making as a flexible, emergent process, grounded in physical and material engagement, that always invites innovation and adaptation to a particular context and practical purpose. The ontological perspective reflected here is an understanding of a world in which everything is interconnected and in a process of change.

If the hylomorphic model is about following a series of steps that lead to the pre-planned object, the alternative is to follow:

“… a passage along a path in which every step grows from the one before and into the one following on an itinerary that always overshoots its destinations … (M) aking is a journey; the maker a journeyman and the essential characteristic of his activity is not that it is concatenated but that it flows (Ingold, 2013, p. 45).

The maker is part of the world and what is made. The maker must engage with the material, and the material will engage with the maker. There involves a process of “´surrendering` to the material and then ´following where it leads’” (Ingold, 2013, p. 45). Bergson argued that this kind of creative, emergent process comprises a central aspect of human experience (Kreps, 2015); other writers have described it as “bringing forth” the possibilities inherent in a situation (Varela et al., 2016; Rolla and Figueiredo, 2021). What all this means is that realizing the possibilities associated with the notion of therapy as making, requires intellectual engagement in unpicking and undoing the hold that an implicit hylomorphism has exerted over psychotherapy theory, research and practice.

### Co-producing

Psychotherapy comprises a particular and distinctive context for making. Most writers on embodied, emergent making have tended to use examples that involve a single person acting on an inert substance, such as an artisan creating a piece of pottery on a wheel (Malafouris et al., 2014; Brinck and Reddy, 2020). By contrast, an essential quality of therapy as making is participation in a process of *co-producing*: therapist and client are working together on whatever it is that is being made. In using the concept of co-producing, we wish to draw a distinction between what we are describing, and the more widely-used concept of collaboration. We believe that, to a large extent, and despite notable exceptions such as Anderson (1996), the concept of collaboration has been used in psychotherapy to refer to situations in which the client actively commits to, and participates in, a therapeutic plan that is ultimately grounded in the theoretical stance of the therapist. By using terms such as co-producing, we wish to open up a space for consideration of a process that draws not only on the theory and practical experience held by the therapist, but also—and to an equal extent—on the experience and knowledge of the client.

The co-production process of making that occurs within psychotherapy can be viewed as encompassing three interconnected threads; rhythm, materiality and metacommunication.

A crucial aspect of maintain rhythm in any co-produced process of making is what developmental psychologists (Bateson, 1979; Stern, 1985; Malloch and Trevarthen, 2009) describe as turn-taking: the rhythmic shift of responses between two or more participants. This kind of rhythmic dance or musicality between two persons is a means of joining together. Ingold (2017) uses the term “interstitial differentiation” to describe this process: “…the way in which difference continually arises within the midst of joining *with*, in the ongoing sympathy of going along together” (Ingold, 2017, cursive in the original text). The experience of a going along together carries a sense of “doubleness”: being a distinct person and at the same time being joined with the other.

Ingold (2018) regards rhythm/turn-taking as part of a broader process of *correspondence*. His use of the concept of correspondence here does not refer to the more traditional meaning where we match one set of elements or objects with another by some principle of similarity between what is matched. Instead, correspondence is understood as “…the process by which beings or things quite literally co-respond or answer to one another over time, as for example in the exchange of letters or of words in conversation” (Ingold, 2018, p. 26). Correspondence (the capacity to be responsive to each other, or *co-respond*) is a central aspect of how we relate to each other, and the experience of togetherness (Sundet and Torsteinsson, 2009). It is about attending to each other as we go along together, and about how we adjust our responses to each other to accommodate our goals, preferences interests and ideas about the good life. It is about attending to one another, as we go along together: “…every being finds its singular voice in the sharing of experience with others” (Ingold, 2018, p. 26). Correspondence then is a rhythmic process that is open-ended, dialogical and attuned to the not yet known, the new and unique (Ingold, 2021).

As well as on-going flow of co-respondence, the act of making-together draws on materiality and metacommunication. Correspondence between persons inevitably involves attention to materiality, the embodied nature of the interaction, such as movement, posture, gesture, voice quality, breath patterns, gaze, smell, visceral phenomena, and many other factors. It is shaped by, and makes use of, what is afforded by the material environment in which interaction takes place, including clothing, furnishing, sounds, objects, landscapes, and living things. The concept of metacommunication refer to the way in which human interaction is given meaning in relation to the purpose or goals of the encounter. Bateson (2000) proposed that joint action always takes place within a system of meanings that allow participants to evoke higher-order levels of meaning to modify or clarify their understanding of what is directly happening. For example, two children are observed wrestling with each other. To understand the meaning of that episode, it is necessary to pay attention to cues that indicate either that “this is play” or “this is serious.” Similarly, the content of a conversation between a therapist and client takes place within a frame defined by metacommunication that “this is confidential” and “this is therapy.” The same dialogue would be understood quite differently in a situation that was framed as a friend-to-friend conversation.

We suggest that most therapists and clients already appreciate and recognize the relevance of the various processes described above: back-and-forth co-responding, the experience of rhythm and energy in a session, the influence of bodies and objects, and the importance of standing back to look at whether what is happening is aligned to an overarching purpose of the therapy. None of what was written above should come as a surprise. However, at the present time these aspects of practice are given little attention within the dominant interventionist perspective on therapy and within mainstream therapy theory. For example, there are few examples of studies that directly document or analyze therapist-client co-production (Hartogs et al., 2013; Blunden, 2021; Klevan et al., 2021; Råbu and Moltu, 2021).

### Making as connecting

The adoption of a therapy as making-together perspective invites consideration of the intrinsic relational dimension of any type of making. The work of sociologist David Gauntlett has provided us with valuable ways of thinking about these questions. Although his writing and research has primarily focused on the social effects of the internet and social media, these topics led Gauntlett (2018) toward an interest in how ordinary people used these communication channels to make blogs and websites that allowed them to express themselves and share ideas with other like-minded individuals. This topic made him curious about pre-internet activities that provided similar opportunities for personal creativity, such as craft-making, music-making, cooking and even building structure from Lego bricks. What Gauntlett (2018) does is to look at the everyday experience of correspondence in the context of forms of making that are familiar to most people.

A central theme in Gauntlett (2018) analysis of making is that it inevitably involves a process of connecting with others. Indeed, for Gauntlett, making *is* connecting:

- making something new requires connecting things together (materials, ideas, or both);
- acts of creativity usually involve, at some point, a social dimension and that connects the maker with other people;
- making and sharing things increases the engagement and connection of the maker with their social and physical environments. (Gauntlett, 2018, p. 10).

It is important to understand that Gauntlett (2018) uses “creative” to refer to everyday acts of making, rather than the activities of elite-level creative artists. An example is the process of baking a birthday cake for a friend or family member. Throughout the process the baker needs to make connections between the design of the cake and the preferences of its recipient, between the recipe as written down in a book and the ingredients that are available to them, and so on. The act of baking may involve connecting with other people in terms of remembering the words of whoever taught them to bake, and consulting others about details of the recipe or when the final product might need to be delivered. Finally, the cake itself is a gift, and the center-piece of a family gathering. The quality of connection contributes to the quality of the final product. The act of engaging in a task that requires presence and mindfulness provides an opportunity for self-expression and strengthen relationships. These different facets of making have important implications for how we understand psychotherapy as a process of making.

Unlike the usual experience of baking, therapy involves constant interaction between therapist and client. Multiple connections are being made between how each participant thinks and feels about the topic being explored. Connections are also made between memories, present moment experiencing, and imagined futures. Shared understanding and action plans that are co-produced on the basis of the knowledge and experience of both therapist and client. Gauntlett (2018) suggests that when a person makes something it is not just to realize an idea that they started out with (the hylomorphic model), “…but rather a process of discovery and having ideas *through* the process of making. In particular, taking *time* to make something … gave people the opportunity to clarify thoughts or feelings, and to see the matter in a new light” (Gauntlett, 2018, p. 12). There is evidence that, compared to reflecting solely on a topic, reflecting while engaged in a relevant task (i.e., active making) can facilitate a deeper level of engagement and understanding (Culpepper and Gauntlett, 2021; Shirota, 2021).

The second and third principles of making-as-connecting identified by Gauntlett (2018) highlight ways in which making something creates connections with other people who have similar experiences or knowledge, and the impacts that the thing-that-is-made has on others. These aspects of connection refer to relational possibilities within the everyday life of the client that are frequently neglected in therapies that emphasize the significance of therapist-initiated interventions. In the example of Alec, described earlier, the client found that other people he knew had also been affected by panic attacks, and were happy to discuss the strategies that they had found helpful or unhelpful in relation so such experiences. In addition, he used his newly-developed understanding of key episodes in his life, worked out in therapy sessions, to repair his relationship with his father, who then became an important source of practical and emotional support.

## Implications for therapy practice

The idea that psychotherapy can be understood as a process of making invites new ways of thinking about theory, research and practice. The full implications of adopting this perspective – including its limitations - will only become clear over time, as it is adopted by practitioners in different contexts and with different interests. Nevertheless, we believe that at this stage, it is possible to glimpse some of the ways in which a making perspective might lead to a revisioning of therapy. Implications for practice of a making-oriented perspective include: how we talk about in therapy sessions, the nature of therapy training, and establishing dialogue between psychotherapists and colleagues in other disciplines and occupations.

### How we talk in therapy sessions

A position that views psychotherapy as a process of making-together is reflected in the way that client and therapist talk about their work together. As well as the usual conversations around the client’s problems and what has happened between one session and the next, a making perspective necessitates on-going dialogue around collaborative planning and decision-making, focusing on topics as what the client wants to use therapy to achieve, how the skills and knowledge of each participant can be combined to enable this happen, and the adequacy of what is being made in relation to whatever it is that the client might want to take away from therapy. This involves naming what is being made, and specifying how it will function in the person’s life: “if I can *build a more adequate understanding of how my parents’ alcoholism shaped the way I think about myself and relate to people*, then I will be able to begin to get closer to other people.” It also involves talking about how the process of making will be organized: “I realize that one of the things I need to do, to build an understanding of how my parents’ drinking influenced how I am as a person, is to start to talk about some of the awful memories I have.” As with any situation in which people work together to make something, there is a rhythm to the work, that moves back and forward between directly engaging in therapy tasks and activities, and standing back to metacommunicate in order to develop a shared vision of what is being made and how to co-ordinate or align the inputs of each participant to that end.

The language of psychotherapy theory is largely dominated by nouns that describe entities – self, unconscious, cognition, empathy - that are reified by researchers in the form of variables (e.g., working alliance). By contrast, adopting a psychotherapy as making perspective invites an action-based language that involves more use of verbs and …*ing* words. This kind of shift was anticipated and advocated in the 1970s by the psychoanalyst Roy Schafer (1976) and in the writings of the interdisciplinary scholar Gregory Bateson (2000). The rationale for the use of action language is that it conveys a sense of the person as an active and purposeful agent who is constantly responding to a world that is changing. By contrast, human intentionality and agency get lost in a language of nouns and variables, with the risk of generating totalizing descriptions (e.g., a schizophrenic, a borderline) that do not acknowledge the fluidity and multiplicity of persons. Some psychotherapists have argued against the adoption of action language, on the grounds that it may suppress or negate the use of entity-based metaphors (I feel heavy”; “I am like a machine”) that are meaningful to people and important information about their lived experience (Spence, 1982). In response, we would suggest that the everyday language used by clients to talk about their problems is saturated with action language (hurting, coping, coming to terms with, getting a handle on, moving on, going round in circles…) and that it is the language of contemporary professional discourse that limits what is being said. In supporting greater use of action language, a making perspective allows clinicians to get closer to important aspects of the lived experience of the client (Larner, 2015).

Increased attention to action-oriented and agentic ways of talking has important implications for research in psychotherapy. For example, research on how to understand the client-therapist relationship feeds into therapy practice and training. The dominant tradition has been research into the working alliance, and its constituent elements of the strength of the therapist-client bond and their agreement over goals and tasks. While this body of research has undoubtedly made a valuable contribution to training and practice, it has become apparent to many researchers that further practical insights require moving beyond group-based analyses of scores on measures of static entities, and looking instead at what the client and therapist are actually doing. An example of this way of thinking and talking can be found in a study by Oddli and Rønnestad (2012) that identified what experienced therapists do in early sessions to build a relationship with their clients, such as explor*ing* the clients’ solution strategies, shar*ing* the basis for decisions about the direction of therapy, and educat*ing* the client in relation to mechanisms of change.

A further aspect of therapeutic conversations that is highlighted by a making perspective, is the importance of being willing to talk about causality. Hylomorphism has influenced the way that therapy is practiced in that it has led to practitioners and researchers being wary around the concept of causality. Hylomorphism assumes that events are shaped by the operation of underlying forms not immediately observable. As a consequence, identifying ultimate causes is a complex and demanding process, that requires the application of a rigorous method of inquiry. These methods were outlined by the philosopher David Hume (1711–1776), who argued that to establish causality it is necessary to collect multiple observations that demonstrate that the cause always closely precedes the effect. Hume’s criteria for determining causes (time asymmetry, contiguity, and constant conjunction) have had a crucial impact on the development of experimental methods in science (Hume, 1978/1739). They have influenced psychotherapy research and practice through the widely-held assumption that, to be credible, causal statements need to be backed up by evidence from randomized controlled trials (RCTs). From the point of view of the lived experience of both therapists and clients, this way of thinking about causality encompasses unhelpful assumptions. In everyday life, and in therapy, it is clear that a single event may have massive causal consequences – the requirement that repeated occurrences need to be observed does not always hold. In addition, in everyday situations, things are caused by multiple interacting influences: the abstract ‘billiard ball’ scenario used by Hume to illustrate his model, almost never applies.

In the non-hylomorphic real world, everything is interconnected. Navigating one’s way through everyday life is based on an awareness of a multiplicity of possible causal links and sequences. This is particularly the case in situations of making. For instance, when baking a cake it is necessary to understand the causal effect of heat on cake ingredients. Similarly, in therapy, clients and therapists are interested in the extent to which the activities and methods they pursue are likely to have an intended causal effect: Will being more open to my feelings lead to being less depressed? An important characteristic of the experience of therapy is that it can often involve acting in new ways – a client who is depressed may have always suppressed their feelings, and as a result will have little basis for being able to know what kind of effect being more open to feelings will have on them (Paul and Healy, 2018). An important strategy for learning about ‘what will work for me’ in therapy is to develop a causal explanation of how one’s problems developed, and what has been successful (or not) in the past in relation to managing them (Carter et al., 2017; Midgley et al., 2017; Sørensen et al., 2020). Another strategy is to use time in therapy to talk about causality and develop a shared causal understanding (Eells et al., 2011; Newman et al., 2013; Larkings et al., 2019, 2021).

In terms of building a capacity to support clients to be aware of implicit causal explanations that might be maintaining their problems and inhibiting their efforts to make a more satisfactory life, and identify productive causal sequences in which they might invest effort, it is helpful for therapists committed to a making stance to develop a differentiated appreciation of the nature of everyday pragmatic reasoning. Many valuable insights are available in the literature on functional analysis of behavior (Haynes et al., 2012). The concept of affordances, initially developed by the environment psychologist J.J. Gibson (2014), provides a further useful perspective (Costall, 1995; Bohart, 2007). Rather than viewing one event causing another, an affordance approach regards the initial event or situation as opening up (affording) a set of possibilities. For example, a client expressing a painful emotion opens up many possible responses on the part of the therapist: they might make an empathic response, say, nothing, or confirm a diagnosis. A similar philosophical perspective on causality approach involves thinking about entities as possessing causal powers or dispositions (Mumford and Anjum, 2011). For example, a Rogerian therapist has a disposition toward making an empathic response, whereas a psychiatrist is disposed to collect diagnostic information. All of these ways of thinking about causality are grounded in an assumption of an interconnected reality in which everything is already in a process of change.

A psychotherapy as making-together perspective highlights the importance of developing strategies for facilitating the process of talking about causality. This can involve unpacking the causal significance and meaning of topics such as client preferences (Norcross and Cooper, 2021), and developing visual mapping techniques such as timelines (McLeod and McLeod, 2022) and vector diagrams (Low, 2017) to facilitate collaborative exploration of causal sequences.

A final conversational process associated with a making perspective is *theorizing*. Making sense, and understanding, represent an important category of making that occurs in therapy. Many clients want to make sense of how their problems have developed, in order to anticipate and avoid situations that might trigger these patients in future. Clients may wish to understand how and why introducing a new activity into their life, such as mindfulness or being more assertive, might be expected to have a positive effect. These (and other) types of making can be seen as constituting a process of active theorizing: using available ideas to construct a framework of understanding that gives meaning to experience and opens up opportunities for moving in the direction of more satisfying and productive action. The concept of theorizing can be contrasted with the construction and application of formal theory. The latter comprises an essentially hylomorphic approach, in terms of specifying a set of propositions taken to be known to be true, and are then used to predict or determine the best course of action in any particular situation. By contrast, everyday or situated theorizing involves weaving together ideas, constructs, metaphors and propositions that are locally available, to create a way of seeing and understanding that is pragmatically useful (Swedberg, 2016a; Swedberg, 2016b). In relation to the areas of experience that clients and therapists are working together to make sense of, each participant is likely to have multiple theoretical perspectives at their disposal. For instance, clients are likely to be familiar with basic concepts of therapy theories that have been assimilated into popular culture, such as psychoanalysis, CBT and the ideas of Carl Rogers. They are also likely to be able to draw on general scientific concepts such as genetics, common-sense ideas such as willpower, and religious ideas such as karma.

### Training

A therapy as making perspective invites consideration of how counselors and psychotherapists can be supported to acquire confidence and competence in such an approach in professional training, and to deepen such a stance over the course of their career. A making perspective can be introduced in training by describing and talking about it, providing examples of what it looks like in relation to practice, and exercises in which trainees reflect on experiences of making and co-production in their own lives. Further possibilities include making use of ideas and learning activities from the arts, and from the field of design. A central element in the kind of making that we have outlined in the present paper, is an open-ness to new ways of approaching a client’s problems and goals, and the construction of a space in which the client’s ideas and suggestions can have an equal influence. In music and theatre this kind of activity is described as improvisation (Nachmanovitch, 2019). Experiential learning activities adapted from theatre and music training can be used in therapist training to develop skills and awareness around ways of facilitating co-production, embodied interplay and mutual empathy between client and therapist, and capacity to stay in the present moment (Bayne and Jangha, 2016; Kelly et al., 2019; Romanelli and Tishby, 2019). For example, in improvisation exercises in theatre school, actors are encouraged to view the actions and statements of their impro partners as “offerings” that can be taken up and further articulated (Johnstone, 1979). Within medical and healthcare education, training in design thinking (Brown, 2009) has been adopted as means of providing practitioners with skills and strategies that are consistent with a flexible and resourceful maker approach to complex problems (McLaughlin et al., 2019; Sandars and Goh, 2020). The relevance of design thinking for psychotherapy practice has been explored by von Thienen and Meinel (2014) and von Thienen et al. (2017). Improvisation and design thinking comprise two particular examples of how a broader capacity to “think like an artist” may be useful for therapists (Gompertz, 2015). There is evidence that some therapists already make use of this way of thinking and seeing to enhance their work with clients (for example, in Råbu et al. (2016)).

### Dialogue with colleagues in other disciplines and occupations

The assumption that psychotherapy is based on delivering interventions arising from pre-established theory, has resulted in a tendency for therapy training, research and general professional discourse to be largely restricted to sources within the field of psychology. This means, in effect, that when looking for ways to enhance their practice, therapists mainly talk to other therapists. One of the most significant implications of adopting a therapy as making and co-producing perspective is that it can lead to productive dialogue with colleagues in other disciplines and occupations who are moving in the same direction. Many of the sources cited in the present paper have been drawn from disciplines such as philosophy, the arts and humanities, political science, history, archaeology, sociology and social anthropology. The notion of making as an emergent embodied activity has been explored in relation to fields of practice such as archaeology, design and music (Anusas and Ingold, 2013; Ingold, 2013; Gürsoy, 2016; Payne and Schuiling, 2017), medicine (Baruch, 2017) and broader changes in patterns of working life within society as a whole in the direction of reclaiming craft traditions (Sennett, 2008) and what has been characterized as the “maker movement” (Dougherty, 2016; Marotta, 2021).

## Conclusion

The events and experiences that move us, that make an enduring difference to our lives, do not come from the application of theories and systems of thought. What we remember are the occasions when something is made in the moment, as a result of a fundamental human capacity for connection and working together. The origins of all therapy theories and protocols can be traced back to such moments of discovery. Such theories represent valuable ways in which such insights can be shared and disseminated. However, the implementation of theories and protocols in the absence of an appreciation of the open edge of knowing has the effect of reinforcing the use of pre-determined intervention, and diminishing the perceived value of common knowledge, small things, and the capacity for co-construction. We need something more, in addition to theory-specific therapies. It is necessary to be willing to meet the unexpected, or not-before-met perspective, event or practice of living. The world is richer and more complex than our theories. As a result, we need an understanding of psychotherapy that is open towards the radically new, the not-met and unknown, and the given (Marion, 2002).

A key aim of the present paper has been to elucidate the idea of psychotherapy as making in relation to the question of what are the basic ingredients or elements from which the eventual products of therapy are constructed. We hope that we have provided a starting point for further exploration of this question, in relation to an appreciation of the deeply-ingrained cultural skills and knowledge, and ways of talking, that make it possible for therapists to work together to address everyday life difficulties, and a framework for making sense of how this kind of process can unfold. A crucial limitation of the paper lies in the fragmentary and subjective nature of the case examples that we were able to provide. A more comprehensive understanding of the implications and possibilities arising from an image of psychotherapy as making, requires research and practice examples that explore the interactive, co-produced and contextualized studies and of making processes in routine practice, along the lines of Hartogs et al. (2013) and Råbu and Moltu (2021).

## Data availability statement

The original contributions presented in the study are included in the article/supplementary material, further inquiries can be directed to the corresponding author.

## Author contributions

All authors listed have made a substantial, direct, and intellectual contribution to the work and approved it for publication.

## Conflict of interest

The authors declare that the research was conducted in the absence of any commercial or financial relationships that could be construed as a potential conflict of interest.

## Publisher’s note

All claims expressed in this article are solely those of the authors and do not necessarily represent those of their affiliated organizations, or those of the publisher, the editors and the reviewers. Any product that may be evaluated in this article, or claim that may be made by its manufacturer, is not guaranteed or endorsed by the publisher.
